# Mental and physical training with meditation and aerobic exercise improved mental health and well-being in teachers during the COVID-19 pandemic

**DOI:** 10.3389/fnhum.2022.847301

**Published:** 2022-08-23

**Authors:** Docia L. Demmin, Steven M. Silverstein, Tracey J. Shors

**Affiliations:** ^1^Department of Psychology, Center for Collaborative Neuroscience, Rutgers University, Piscataway, NJ, United States; ^2^Department of Psychiatry, Rutgers University, Piscataway, NJ, United States; ^3^Department of Neuroscience and Ophthalmology, University of Rochester, Rochester, NY, United States

**Keywords:** COVID-19, stress, mental health, aerobic exercise, mindfulness

## Abstract

Teachers face significant stressors in relation to their work, placing them at increased risk for burnout and attrition. The COVID-19 pandemic has brought about additional challenges, resulting in an even greater burden. Thus, strategies for reducing stress that can be delivered virtually are likely to benefit this population. Mental and Physical (MAP) Training combines meditation with aerobic exercise and has resulted in positive mental and physical health outcomes in both clinical and subclinical populations. The aim of this pilot study was to evaluate the feasibility and potential effectiveness of virtual MAP Training on reducing stress and improving mood and well-being in teachers. Participants (*n* = 104) were from recruited online from kindergarten to grade twelve (K-12) schools in the Northeastern region of the United States and randomly assigned to a 6-week program of virtual MAP Training (*n* = 58) or no training (*n* = 13). Primary outcomes included pre-intervention and post-intervention ratings on self-report measures of social and emotional health. Changes in cognitive functioning and physical health were also examined in secondary analyses. By intervention end, participants in the MAP Training group reported less anxiety and work-related stress compared to those who received no training (*d*s = −0.75 to −0.78). Additionally, MAP Training participants reported improvements in depressive symptoms, rumination, work-related quality of life, perceived stress, and self-compassion (*d*s = 0.38 to −0.82), whereas no changes were observed in the no training group. Participants also reported increased subjective ratings of executive functioning, working memory, cognitive flexibility, and fewer sleep disturbances (*d*s = −0.41 to −0.74). Together, these results suggest that the combination of meditation and aerobic exercise is an effective virtual intervention for improving mental health and well-being among K-12 teachers and may enhance resilience to stressful life events such as occurred during the coronavirus pandemic.

## Introduction

The coronavirus disease 2019 (COVID-19) pandemic upended education systems nationwide and created a uniquely stressful and demanding situation for teachers. Teaching has long been recognized as a high-stress profession, with 46% of teachers reporting high daily stress during the school year (Gallup, 2014). Teacher stress has been linked to high job demands (McCarthy, 2019), as educators struggle to balance pressures from administrators, students, and parents. Additional sources of stress include a perceived lack of support, poor working conditions, and student misbehavior (Shernoff et al., 2011; Richards, 2012). Together, these factors contribute to low job satisfaction (Liu and Ramsey, 2008; McCarthy, 2019), reduced occupational commitment (McCarthy, 2019; Fitchett et al., 2021), and high rates of attrition (Boe et al., 2008; Conley and You, 2009). Teachers are also likely to experience workplace fatigue (Fitchett et al., 2021) and burnout (Haberman, 2005; Bottiani et al., 2019) as a result of work-related stress. Moreover, job stress has been associated with mental health symptoms including anxiety, depression, and somatization (Godin et al., 2005; Mark and Smith, 2012), as well as physical health effects such as increased disease risk, weight gain, and poor sleep (Bosma et al., 1998; Kivimäki et al., 2006; Knudsen et al., 2007).

The onset of the COVID-19 pandemic exacerbated some of the mental health outcomes associated with this high-stress occupation. For example, increases in anxiety and depressive symptoms following the onset of the pandemic have been reported globally (Ciacchella et al., 2022). Early studies suggest a high percentage of educators have experienced significant distress (Aperribai et al., 2020; Ozamiz-Etxebarria et al., 2021) as well as reduced quality of life (Lizana et al., 2021). Moreover, teachers have reported moderate levels of secondary traumatic stress (i.e., avoidance, intrusion, arousal; Anderson et al., 2021). Importantly, concerns about health and safety, teaching demands, parent communication, and administrative support were identified as significant predictors of teacher burnout-stress (Pressley, 2021). Further, rates of teacher attrition are projected to increase with COVID-19 cited among the top reasons teachers chose to leave the profession in 2020 (Diliberti et al., 2021). Thus, the COVID-19 outbreak placed teachers in critical need of mental health support.

Mindfulness is often defined as becoming aware of what you are sensing and feeling in the present moment, without judgment or interpretation and is usually practiced during sitting and/or breathing meditation. In recent years, there has been growing interest in incorporating mindfulness training with meditation into schools. Much of this work has focused on students rather than teachers, with modest increases in student learning, cognition, and psychological well-being [see Zenner et al. (2014) and Carsley et al. (2018) for review]. Meta-analyses suggest that mindfulness-based interventions may provide additional benefit (Klingbeil and Renshaw, 2018; Zarate et al., 2019). For example, studies report large increases in self-compassion in teachers (Roeser et al., 2013; Frank et al., 2015), along with moderate decreases in stress and anxiety, and small, but significant, improvements in depression and burnout (Zarate et al., 2019), and medium effects overall (Klingbeil and Renshaw, 2018). Positive effects on physical health have also been reported in teachers, such as improvements in sleep quality and decreases in fatigue (Crain et al., 2017; Dave et al., 2020). Teachers who engaged in the most established meditation-based intervention, Mindfulness-Based Stress Reduction (MBSR), reported increases in mindfulness and sustained attention (Flook et al., 2013). Thus, mindfulness training through meditation may be an effective tool for reducing stress and improving health and well-being among teachers, especially while living through a stressful life event as occurred during the coronavirus pandemic.

Aerobic exercise is cardiovascular activity achieved through a large increase in heart rate, usually corresponding to a nearly two-fold increase from the rate at rest. As a result, more oxygenated blood is distributed throughout the body, with a large percentage (∼20%) of it reaching the brain. The benefits of aerobic exercise are widespread, including changes in coronary blood flow, sleep quality, reductions in blood pressure, and systemic inflammation (e.g., Pratley et al., 2000; Hamer et al., 2006; Warburton, 2006; Ismail et al., 2012; Passos et al., 2012; Zheng et al., 2019). But more germane to the present study, regular aerobic exercise is linked to less depression and anxiety (Shamus and Cohen, 2009; Basso and Suzuki, 2017) and greater quality of life (e.g., Pang et al., 2013; Wu et al., 2020). Moreover, exercise is associated with small, but significant, improvements in attention, executive functioning, processing speed, and memory (Smith et al., 2010; Basso and Suzuki, 2017). Whereas a plethora of school-based exercise programs have been developed to promote student engagement, few have examined the potential benefit of exercise interventions for teachers. Abós et al. (2021) investigated the effects of a 16-week physical activity program consisting of playful, strength, aerobic, and back-pain prevention exercises. Teachers who participated in the program reported significant improvements in work-related outcomes such as work satisfaction, vigor, and absorption in comparison to controls (Abós et al., 2021). These findings, together with substantial evidence of mental and physical health benefits, suggest that exercise interventions may promote positive outcomes in teachers.

In this pilot study, we delivered a brain fitness program that combines mental training with meditation and physical training with aerobic exercise to teachers during the COVID-19 pandemic. The program, known as MAP Training, includes 30 min of focused-attention and slow-walking meditation, both done in complete silence. These activities are immediately followed with 30 min of aerobic exercise (Shors et al., 2014; Shors, 2021). In clinical and subclinical populations, MAP Training has yielded positive effects on mental health with decreases in depression, rumination, and post-traumatic thoughts, along with an increase in quality of life (Shors et al., 2014, 2017, 2018; Alderman et al., 2016; Lavadera et al., 2020). In addition, studies suggest an increase in the volume of oxygen consumption as measured with VO_2_ (Shors et al., 2014), synchronous brain activity as measured with electroencephalography (EEG) during cognitive control (Alderman et al., 2016), and discrimination learning during a pattern separation task associated with neurogenesis in the adult hippocampus (Millon et al., 2022). Additionally, engaging in the combination of mental and physical training activities has been shown to be especially effective when compared to engaging in one activity on its own (Shors et al., 2018).

During the coronavirus pandemic, there was increased need for interventions and exercise programs that could be delivered and practiced online through virtual mechanisms such as Zoom. To meet this need, we evaluated the feasibility of virtual MAP Training on reducing stress and improving psychological, cognitive, and health outcomes in primary and secondary school teachers who were living through the COVID-19 pandemic.

## Materials and methods

### Participants

Participants included K-12 (kindergarten through grade 12) educators in schools in the states of New York, New Jersey, Connecticut, and Pennsylvania, given that the impact of COVID-19 was similar among these regions (i.e., containment strategies, case counts). Subjects were recruited in three waves (from June 2020 to July 2020) through flyers distributed to area school administration (e.g., principals, assistant principals) and social media (i.e., Facebook) advertisements. Interested individuals with a physical health condition that may contraindicate vigorous exercise (e.g., history of heart disease, stroke, cardiac arrythmia, uncontrolled asthma, severe joint problems) were excluded from study participation. A computer-generated randomization sequence was used to assign participants to intervention (MAP Training) and waitlist control (No Training) groups using a ratio of 4:1 to obtain a sufficient sample size to test for treatment effects in the MAP Training group. One subject who expressed interest in participating in the study but was unable to attend the MAP Training sessions was thus assigned to the No Training group. The protocol was approved by the Rutgers IRB (Pro2020001365) and electronic informed consent was obtained for each subject prior to participation and reaffirmed at each assessment timepoint.

### Intervention

**FIGURE 1:**
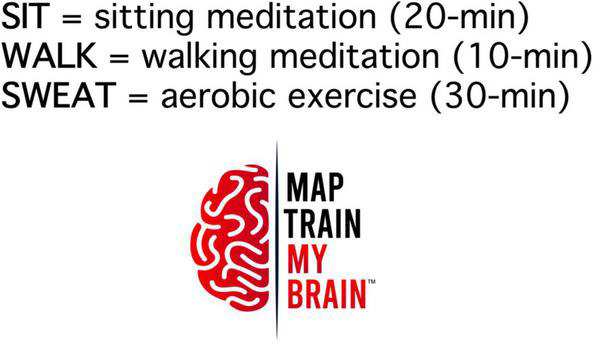
One session of MAP Training begins with 20-min of silent focused attention meditation (SIT), followed by 10-min of silent slow-walking meditation (WALK), and ending with 30-min of aerobic exercise (SWEAT).

Mental and physical Training combines mental training with meditation and physical training with aerobic exercise (Shors et al., 2014; see Figure 1). This program was delivered online *via* the Zoom platform. First, participants were presented with a “brain bit,” which was a short piece of information about the brain to keep them engaged and motivated. Then, they watched and listened as the facilitator instructed them on how to set-up the meditation activities and then engaged in the activities along with the participants. The mental training component consisted of 20-min of a focused-attention (FA) meditation while sitting in silence, followed by 10-min of a walking meditation, again in silence. During the FA meditation, participants were instructed to breathe naturally while bringing their full attention to their breath. They were told to notice the short space between the out-breath and the in-breath (i.e., SIT; Figure 1). Participants were instructed to count the space between each breath, beginning with one and continuing until they lost count. Should their attention wander, they should acknowledge their thoughts without judgement, and return their attention to counting the space between each breath, beginning again with one. A timer was set to ring after 20 min, at which point participants were told to stretch out their legs before standing up. Once they felt ready, they were asked to stand up for the 10-min of walking meditation. During this part of the intervention, participants were instructed to clasp their hands loosely behind their backs and maintain their gaze at the floor ∼3 feet in front of them. They were to focus their attention on their feet while they walked a circular path at a very slow pace, noticing how the weight of the body changes with each step and meanwhile noticing how the bottom of the feet touch the floor (i.e., WALK; Figure 1). As during sitting meditation, participants were instructed to maintain attention on their feet as they walk until they lost concentration, at which time they were to recognize that they have lost their focus of attention and return it to the feet. Again, a timer was set to ring after 10 min. Next, the participants prepared themselves for the physical training component, which consisted of 30-min of moderate intensity aerobic exercise (i.e., SWEAT; Figure 1). Participants began the exercise component with a 5-min warm-up. Next, they were led through a choreographed aerobic exercise routine to popular music. Each session incorporated 9–10 tracks which were rotated in and out each week. The session concluded with a 5-min cool down. Each session was approximately 1 h.

Before training, participants were instructed to take their own heart rate by pressing their finger against the side of their neck and then asked in all sessions, to gauge their heart rate to ensure, to the extent possible, that exercise was performed at a moderate level of intensity. Approximately 20 min into the physical exercise component of each MAP Training session, subjects were directed to count their pulse over a 10 s period, then multiply the value by six. The aerobic range is generally defined as lying between 60 and 80% of participants’ maximum which is calculated by subtracting their age from 220. For most participants, their aerobic range was greater than 90 beats per minute but less than 140.

### Measures

#### Primary outcomes

##### Depressive symptoms

The depression module of the Patient Health Questionnaire (PHQ-9; Spitzer, 1999) was used to evaluate the impact of MAP Training on teachers’ ratings of mood symptoms. The PHQ-9 is a self-report questionnaire consisting of nine items assessing DSM-IV criterion A for a major depressive episode. Participants were asked to rate the frequency of each symptom over the past 2 weeks using a four-point Likert scale ranging from 0 (not at all) to 3 (nearly every day). Total scores can be classified as minimal (0–4), mild (5–9), moderate (10–14), moderately severe (15–19), and severe (20–27). Scores greater than 15 indicate a likely major depressive disorder diagnosis (Kroenke et al., 2001; Kroenke and Spitzer, 2002) and clinically significant change is indicated by a total score reduction of five or more points (Löwe et al., 2004).

##### Anxiety

Anxiety symptoms were measured with the General Anxiety Disorder scale (GAD-7; Spitzer et al., 2006). The GAD-7 is a seven item self-report measure of DSM-IV diagnostic criteria A, B, and C for generalized anxiety disorder. Subjects were asked to rate the frequency of each item over the past 2 weeks using a four-point Likert scale ranging from 0 (not at all) to 3 (nearly every day). The GAD-7 total scores can also be categorized as minimal (0–4), mild (5–9), moderate (10–14), and severe (15–21). Score changes of four points or more on the GAD-7 are considered clinically significant (Toussaint et al., 2020).

##### Ruminative thoughts

The Ruminative Response Scale (RRS; Nolen-Hoeksema and Morrow, 1991) was used to examine the impact of MAP Training on frequency of rumination among teachers. The RRS is a 22-item self-report measure of ruminative thinking. Rumination refers to a pattern of perseverative thinking generally focused on symptoms of distress and their possible causes and consequences and has been closely linked with poor mental health outcomes, especially depression (Nolen-Hoeksema, 1991; Nolen-Hoeksema and Morrow, 1991; Spasojević and Alloy, 2001; McLaughlin and Nolen-Hoeksema, 2011). Participants rated the frequency of ruminative behaviors over the past 2 weeks using a scale from 1 (almost never) to 4 (almost always). The original 22-item RRS used here contains three subscales: depressive, brooding, and reflective. Depressive ruminations focus on mood changes, whereas brooding ruminations tend to focus on negative self-evaluations and judgements involving blame and/or guilt. Reflective ruminations are less tied to mood and more aligned with contemplative thinking and problem solving. Both brooding and reflection have been associated with current depressive symptom severity, whereas reflection has been linked with lower risk for future depression (Treynor et al., 2003; Burwell and Shirk, 2007). However, regardless of subscale, most previous studies with MAP Training report decreases, including participants from clinical and nonclinical populations (Alderman et al., 2016; Shors et al., 2017; Shors et al., 2018; Lavadera et al., 2020; Shors, 2021; Millon et al., 2022).

##### Perceived stress

Subjective evaluations of stress have been associated with severity of depressive symptoms, anxiety symptoms, and experiences of stressful life events (Cohen et al., 1983; Otto et al., 1997). The Perceived Stress Scale (PSS-10; Cohen et al., 1983) was administered to examine the effects of MAP Training on teachers’ experience of stress. The PSS consists of 10 items that assess the degree to which an individual perceives their life to be unpredictable and uncontrollable. Participants were asked to report on their thoughts and feelings over the previous 2 weeks using a five-point Likert scale with scores ranging from 0 (never) to 4 (very often).

##### Quality of life

The Professional Quality of Life Scale (ProQOL; Stamm, 2010) was used to assess the impact of MAP Training on teachers’ quality of life. It has been suggested that in caring for people who have experienced stressful events, caregivers (helpers, etc.) are also at risk of developing stress-related symptoms (Stamm, 1995). The ProQOL is a 30-item self-report questionnaire consisting of three subscales: compassion satisfaction (the converse to compassion fatigue), burnout, and secondary traumatic stress; but given that each subscale is psychometrically unique, capturing both positive and negative outcomes of helping professions, a total score is not recommended (Stamm, 2010). Participants rated the frequency of each experience over the past 2 weeks using a five-point Likert scale ranging from 1 (never) to 5 (very often).

##### Self-compassion

The Self-Compassion Scale Short Form (SCS-SF; Raes et al., 2011) was administered to evaluate the effects of a MAP Training on teachers subjective ratings of self-compassion, which is conceptualized as an openness and non-judgmental understanding of one’s own pain, inadequacies, and failures (Neff, 2003). Participants responded to 12 items assessing frequency of self-compassion over the previous 2 weeks on a five-point Likert scale ranging from 0 (almost never) to 5 (almost always).

##### Distress tolerance

Distress tolerance relates to an individual’s ability to tolerate negative emotional states (Leyro et al., 2010). Low distress tolerance has been associated with the development of mental health problems, such as anxiety (Keough et al., 2010), depression (Lass and Winer, 2020), and posttraumatic stress symptoms (Vujanovic et al., 2011). The Distress Tolerance Scale (DTS; Simons and Gaher, 2005) was administered to examine the effects of MAP Training on teachers’ ability to tolerate negative emotional or aversive states (i.e., distress; Leyro et al., 2010). The DTS is 15-item self-report questionnaire assessing an individual’s perceived ability to tolerate emotions, appraisal of distress, absorption by negative emotions, and regulation of emotions. Participants evaluated their present abilities to tolerate distress using a five-point Likert scale from 1 (strongly agree) to 5 (strongly disagree).

##### Mental and physical health questionnaire

The MAP Health Questionnaire, was included in the assessment battery to further evaluate the potential effectiveness of MAP Training on overall mood and well-being. The questionnaire comprises 20 items derived from four aspects of mental health often measured by existing self-report instruments that assess posttraumatic thoughts (Posttraumatic Cognitions Inventory [PTCI]; Foa et al., 1999) (five items), rumination (RRS; Nolen-Hoeksema, 1991) (five items), anxiety symptoms (Beck Anxiety Inventory [BAI]; Beck et al., 1988) (five items), and depressive symptoms (Beck Depression Inventory [BDI]; Beck, 1961; Beck et al., 1996) (five items). Prior studies of MAP Training in distressed populations indicate that these symptoms are closely linked at baseline and improve with training (i.e., Shors et al., 2018; Shors, 2021; Millon et al., 2022).

#### Secondary outcomes

##### Executive function

Executive functions are a category of mental skill processes that include working memory, cognitive flexibility and self-control of behavior. The Adult Executive Functioning Inventory (ADEXI; Holst and Thorell, 2018) was used to assess perceived changes in these skills. The ADEXI consists of 14 items that comprise subjective estimates of working memory and inhibition. Participants rated their level of agreement with each item over the previous 2 weeks using a five-point Likert scale ranging from 1 (Definitely not true) to 5 (Definitely true).

##### Cognitive flexibility

The impact of MAP Training on subjective estimates of cognitive flexibility was assessed using the Cognitive Flexibility Inventory (CFI; Dennis and Vander Wal, 2010). The CFI is a 20-item self-report instrument consisting of two factors. The Control factor evaluates the extent to which an individual perceives a difficult situation as controllable, and the Alternatives factor measures an individual’s ability to generate multiple explanations and solutions for difficult situations. Participants reported on their cognitive flexibility over the previous 2 weeks using a seven-point Likert scale from 1 (Strongly disagree) to 7 (Strongly agree).

##### Physical health

The Patient Health Questionnaire (PHQ-15; Kroenke et al., 2002) is a self-administered scale for evaluating somatic symptom severity. The PHQ-15 assesses 15 of the most commonly reported somatic complaints in primary care settings (Kroenke, 2003), including gastrointestinal, musculoskeletal, pain, and fatigue symptoms. Participants rated the degree to which they were bothered by each symptom over the previous 2 weeks using a three-point Likert scale ranging from 0 (Not at all) to 2 (Bothered a lot). Total scores can be classified as low (0–5), moderate (6–10), and high (11–15) levels of symptom severity.

##### Sleep quality

The Pittsburgh Sleep Quality Index (PSQI; Buysse et al., 1989) is a commonly used and well-validated research tool for assessing sleep quality (Mollayeva et al., 2016). An abbreviated version of the full scale, the short PSQI (sPSQI), containing 13 of the original 19 items has been developed in an effort to reduce participant burden and increase research utility (Famodu et al., 2018). The sPSQI assesses five components of sleep quality. Sleep latency, sleep duration, sleep efficiency scores are based on reported bedtime, sleep time, wake time, and rise time in the past 2 weeks while sleep disturbances and daytime dysfunction components are rated from 0 (Not in the past 2 weeks) to 3 (Three or more times a week) in terms of frequency. To facilitate scoring, participants were asked to select categorical responses for each of the 13 items.

### Procedures

#### Pre-intervention assessment

All participants completed the initial (pre-intervention) assessment within 1 week prior to the start of the MAP Training sessions. Surveys were administered electronically (i.e., Qualtrics). A unique link was generated for each subject ID and distributed to participants *via* email. The pre-intervention assessment included a sociodemographic and health questionnaire and battery of self-report measures, described above. After completion of the pre-intervention assessment, subjects received a $20 Amazon e-gift card as compensation for their time and participation.

##### Mental and physical training group

Live MAP Training sessions were delivered virtually through the Zoom platform. Recorded MAP Training sessions were accessible with a private YouTube link. Participants in the MAP Training group were asked to engage in one live MAP Training session and one recorded session each week for 6 weeks. Thus, the MAP Training program consisted of two 1-h sessions per week over 6 weeks (12 sessions total). The virtual sessions were led by Dr. Tracey Shors, who developed the MAP Training program (described above). Prior to the initial live session participants were provided with a 30-min video introduction to MAP Training. To maintain participant confidentiality, attendee information (i.e., names, videos) was disabled during the Zoom sessions. At the conclusion of each live MAP Training session, participants were asked to complete a brief Qualtrics survey to assess adherence, which included a multiple-choice question about the content of the live session. We used this to gauge their attendance. We also asked them to report their maximal heart rate during the aerobic exercise component, and indicate their level of engagement (i.e., not very [0–25%], somewhat [26–50%], moderately [51–75%], very [76–90%], fully [>90%]). The survey also asked them whether and if so, how long they had engaged in similar activities during the week and outside of live MAP Training sessions. The survey also asked whether they had completed the weekly recorded session.

##### No training group

Participants allocated to the No Training group did not partake in MAP Training sessions over the course of the study, but were asked each week to report their engagement in meditation and physical activity each week in a Qualtrics survey. They were provided with unlimited access to six recordings of MAP Training sessions at the end of the study.

#### Post-intervention assessment

Within 1 week of the last session, participants in both groups completed the post-intervention assessment consisting of the same set of self-report measures as the pre-intervention assessment (described above). Surveys were distributed electronically and accessed *via* a unique Qualtrics link.

### Data analysis

Analyses were conducted using SPSS Version 27 (IBM Corp, 2020). Between group (MAP Training, No Training) differences on baseline characteristics (i.e., sociodemographic variables [age, race, ethnicity, sex, educational attainment], health history, teaching experience, engagement in mindfulness, and exercise activities) were examined with independent samples *t*-tests and Pearson’s Chi-square tests for independence, where appropriate. An *a priori* power analysis indicated that a total sample size of 99 was necessary to detect large (*f* = 0.50) within-between interaction effects with 90% power (α = 0.05). As a result, the target sample size was 80 for the MAP Training group and 20 for No Training group (4:1 ratio).

A repeated-measures multivariate analyses of variance (MANOVA) tested between-group differences in primary (i.e., psychosocial) and secondary (i.e., cognitive, health) outcomes at pre- vs. post-intervention. Data were assessed for multivariate normality, homogeneity of covariance matrices, and multicollinearity. Significant univariate and multivariate interactions were followed with *post hoc* analyses. Group differences on outcome measures at pre-intervention and post-intervention timepoints were assessed with independent samples *t*-tests, whereas within-group changes were tested with paired samples *t*-tests. In both sets of analyses, a False Discovery Rate (FDR; Benjamini and Hochberg, 1995) correction (α = 0.05) was applied. The effects of MAP Training on primary and secondary outcomes were tested with repeated-measures MANOVAs. Significant univariate and multivariate interactions were followed up with independent and paired samples *t*-tests with an FDR correction applied. Data were further explored with pairwise comparisons, corrected for multiplicity.

#### Missing data

The person mean imputation approach was applied in cases of missing responses to questionnaire items. Reverse-scored items were recoded as needed. Imputed values were then calculated using the mean of the observed item responses for each participant. Mean scores were imputed only for cases in which less than 10% of questionnaire data were missing (i.e., questionnaires with 11 or more items).

## Results

### Participant characteristics

**FIGURE 2:**
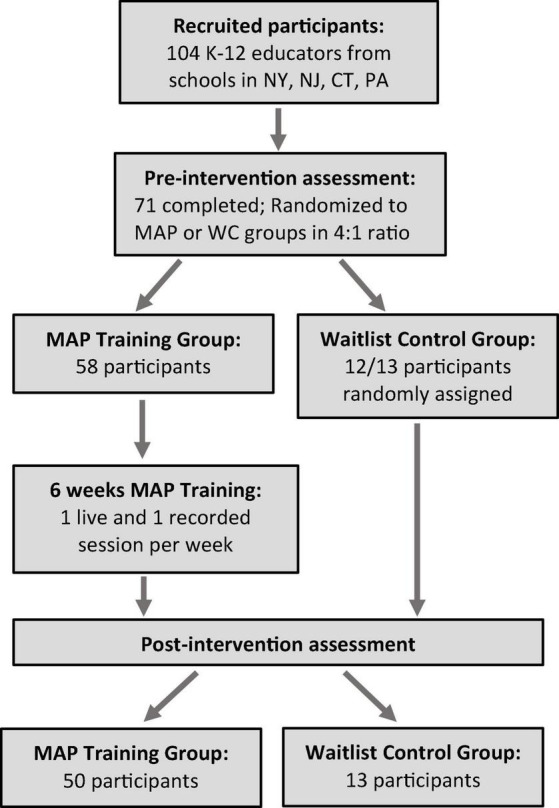
Schematic of participant recruitment, randomization, assessment, and attrition.

Of the 104 teachers recruited, 71 completed the initial baseline assessment and were included in the data analyses. Of these participants, 58 were randomly assigned to the MAP Training group and the remaining participants to the No Training group. One participant was not randomly assigned because they could not attend the live sessions (Figure 2).

Groups did not differ significantly on characteristics prior to training [e.g., age, sex, race, years teaching, experience with mindfulness (including meditation), participation in exercise]. Rates of attrition did not differ by group [χ^2^(1, *N* = 104) = 0.16, *p* > 0.05]. Engagement was assessed after each live session by asking for self-reported engagement and maximal heart rate. On average, participants assigned to the MAP training group engaged in at least four out of the six live sessions and completed approximately half of the six recorded sessions. Based on this information, we considered participants who attended at least four out of six live MAP training sessions (*n* = 35) as treatment-adherent. These treatment-adherent participants and No Training groups did not differ significantly with respect to their characteristics before training (e.g., age, sex, race, years teaching, experience with mindfulness, participation in exercise, etc.; see Table 1). Treatment-adherent and non-adherent participants did not differ on most baseline characteristics, although a greater proportion of teachers in the treatment-adherent group reported no previous experience with mindfulness programs [χ^2^(2, *N* = 58) = 9.09, *p* = 0.01].

**TABLE 1:** Sample characteristics: treatment-adherent MAP training group vs. no training group.

	MAP (*n* = 35)	No training (*n* = 13)	Total (*n* = 48)	Difference
Age (M[SD])	39.3 (10.72)	40.9 (10.3)	39.9 (10.5)	*p* > 0.05
Race (n[%])				*p* > 0.05
Black/African American	1 (2.9%)	1 (7.7%)	2 (4.2%)	
White/Caucasian	32 (91.4%)	11 (84.6%)	43 (89.6%)	
Asian	1 (2.9%)	1 (7.7%)	2 (4.2%)	
Native American/Alaskan	1 (2.9%)	0 (0%)	1 (2.1%)	
Pacific Islander	1 (2.9%)	0 (0%)	1 (2.1%)	
Other	1 (2.9%)	1 (7.7%)	2 (4.2%)	
Hispanic/Latino (n[%])	3 (8.6%)	0 (0%)	3 (6.3%)	*p* > 0.05
Sex (n[%])				*p* > 0.05
Female	33 (94.3%)	12 (92.3%)	45 (93.8%)	
Male	2 (5.7%)	1 (7.7%)	3 (6.3%)	
Known medical condition (n[%])	12 (34.3%)	4 (30.8%)	16 (33.3%)	*p* > 0.05
Type of teaching institution (n[%])				*p* > 0.05
Public	29 (82.9%)	9 (69.2%)	38 (79.2%)	
Private	5 (14.3%)	4 (30.8%)	9 (18.1%)	
Other	1 (2.9%)	0 (0%)	1 (2.1%)	
Years teaching (M[SD])	12.9 (9.0)	14.2 (8.5)	13.2 (8.8)	*p* > 0.05
Teaching Summer 2020 (n[%])	7 (20.0%)	3 (23.1%)	10 (20.8%)	
Experience with mindfulness (n[%])				*p* > 0.05
None	10 (28.6%)	1 (7.7%)	11 (22.9%)	
A little/Some	25 (71.4%)	11 (84.6%)	36 (75.0%)	
A lot	0 (0%)	1 (7.7%)	1 (2.1%)	
Mindfulness past week (n[%])	12 (34.3%)	4 (30.8%)	16 (33.3%)	*p* > 0.05
Meditation	6 (17.1%)	1 (7.7%)	7 (14.6%)	
Deep breathing	7 (20.0%)	3 (2.3%)	10 (20.8%)	
Other	1 (2.9%)	1 (7.7%)	2 (4.2%)	
Exercise past week (n[%])	28 (80.0%)	9 (69.2%)	37 (77.1%)	*p* = 0.05
Aerobic	27 (77.1%)	7 (53.8%	34 (70.8%)	
Strength	9 (25.7%)	5 (38.5%)	14 (29.2%)	
Flexibility	7 (20.0%)	4 (30.8%)	11 (23.0%)	
Balance	3 (8.6%)	1 (7.7%)	4 (8.3%)	
Other	0 (0%)	0 (0%)	0 (0%)	
Total mindfulness (min.) (M[SD])	110.3 (116.1)	159.5 (190.3)	122.0 (136.3)	*p* > 0.05
Total exercise (min.) (M[SD])	762.3 (617.0)	561.7 (504.9)	710.0 (591.1)	*p* > 0.05
MAP engagement^a^ (M[SD])	4 (1)			
MAP HR (M[SD])	129.2 (0.9)			
Live MAP sessions (M[SD])	5 (1)			
Independent MAP sessions (M[SD])	4 (2)			
Total MAP sessions	9 (2)			

^a^1 = Not very (0–25%), 2 = Somewhat (26–50%), 3 = Moderately (51–75%), 4 = Very (76–90%), 5 = Fully (>90%).

### Group differences in primary outcomes

A one-way repeated measures MANOVA tested for significant differences between treatment adherent MAP training participants and those who received no training. The multivariate group × timepoint interaction was not significant (*p* = 0.06). The sample size in the No Training group (*n* = 11) was less than the number of dependent variables in the analysis (*n* = 12), and thus the analysis may have been underpowered. Nevertheless, a series of one-way ANOVA’s on these variables revealed a significant group x timepoint interaction on multiple variables, indicating that the MAP group demonstrated greater change than the No Training group on each of the following outcomes: overall mood and well-being [MAP Health Questionnaire; *F*_(1,43)_ = 5.40, *p* = 0.02, η^2^ = 0.11], depressive symptoms [PHQ-9; *F*_(1,43)_ = 6.34, *p* = 0.02, η^2^ = 0.13], anxiety symptoms [GAD-7; *F*_(1,43)_ = 13.15, *p* < 0.01, η^2^ = 0.23], secondary traumatic stress related to profession [ProQoL-Secondary Traumatic Stress; *F*_(1,43)_ = 7.74, *p* = 0.01, η^2^ = 0.15], and perceived stress [PSS-10; *F*_(1,43)_ = 7.97, *p* = 0.01, η^2^ = 0.16]. A series of independent samples *t*-tests compared groups on psychosocial outcomes at each timepoint, with no significant differences at baseline (*p*s > 0.05). Post-intervention scores were significantly different between groups with respect to anxiety [GAD-7; *t*(46) = −2.40, *p* = 0.02, *d* = −0.78] and professional quality of life related to experiences of secondary traumatic stress [ProQoL-Secondary Traumatic Stress; *t*(46) = −2.31, *p* = 0.03, *d* = −0.75], with participants in the MAP Training group reporting lower levels of anxiety and less work-related stress than the No Training group, although between-group differences were not signficant after adjusting for multiple comparisons.

**FIGURE 3:**
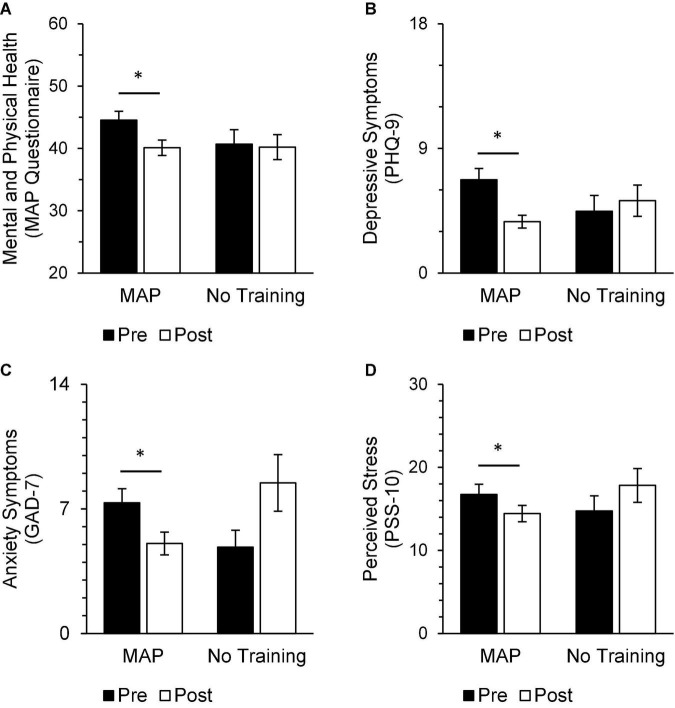
In the MAP group, post-intervention scores were significantly improved from pre-intervention scores on measures of trauma and mood [**(A)** MAP Health Questionnaire], depressive symptoms [**(B)** PHQ-9], anxiety symptoms [**(C)** GAD-7], and perceived stress [**(D)** PSS-10]. There were no differences between pre-intervention and post-intervention scores on these same measures in the No Training group and no differences between groups at either timepoint. Asterisks indicate significant adjusted *p*-values. Scores range from 20 to 80 on the MAP Health Questionnaire, 0 to 27 on the PHQ-9, 0 to 21 on the GAD-7, and 0 to 40 on the PSS-10.

**FIGURE 4:**
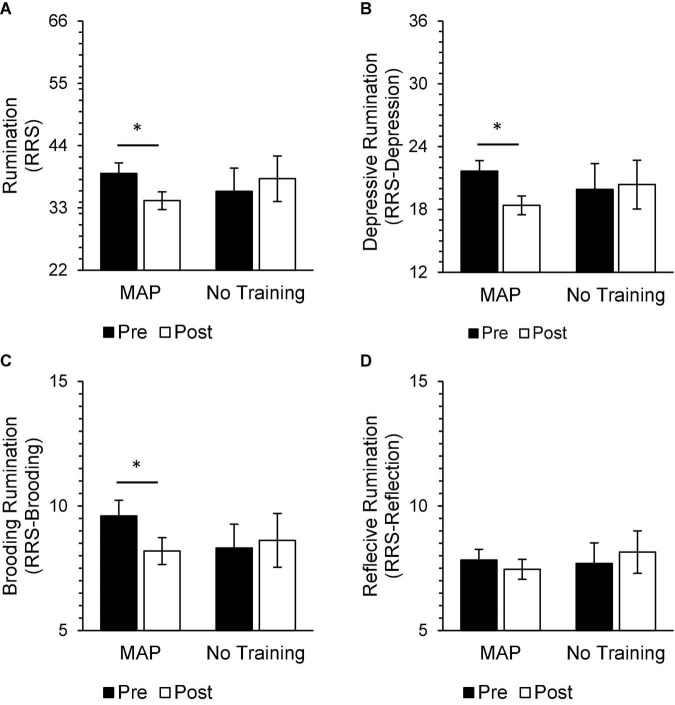
In the MAP group, post-intervention scores were significantly improved from pre-intervention scores on measures of rumination [**(A)** RRS], including depressive rumination [**(B)** RRS-Depression] and brooding [**(C)** RRS-Brooding], but not reflective rumination [**(D)** RRS-Reflection]. There were no significant differences between pre-intervention and post-intervention scores on these same measures in the No Training group. There were also no significant differences between groups at either timepoint. Asterisks indicate significant adjusted *p*-values. Total scores on the RRS range from 22 to 88, RRS-Depression subscores range from 12 to 48, RRS-Brooding subscores range from 5 to 20, and RRS-Reflection subscores range from 5 to 20.

**FIGURE 5:**
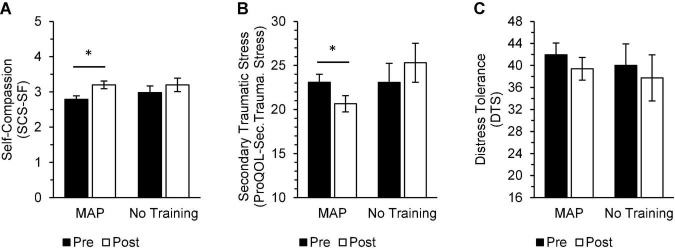
In the MAP group, post-intervention scores were significantly improved from pre-intervention scores on measures of self-compassion [**(A)** SCS-SF] and secondary traumatic stress related to profession [**(B)** ProQoL-Secondary Traumatic Stress], while there were no significant differences between pre-intervention and post-intervention scores on these same measures in the No Training group. There were no significant within-group differences in distress tolerance [**(C)** DTS]. There were no significant differences between groups at either timepoint. Asterisks indicate significant adjusted *p*-values. A mean is calculated for the SCS-SF. Scores on the DTS range from 16 to 80.

In the treatment-adherent MAP Training group, scores after the intervention significantly improved on measures of mood and well-being [MAP Health Questionnaire; *t*(34) = 3.82, *p* < 0.01, *d* = 0.65], depressive symptoms [PHQ-9; *t*(33) = 3.82, *p* < 0.01, *d* = 0.66], anxiety symptoms [GAD-7; *t*(34) = 3.36, *p* < 0.01, *d* = 0.57], rumination [RRS; *t*(34) = 2.91, *p* = 0.01, *d* = 0.49], brooding [RRS-Brooding; *t*(34) = 2.54, *p* = 0.016, d = 0.43], depressive ruminations [RRS-Depression; *t*(34) = 3.20, *p* < 0.01, *d* = 0.54], secondary traumatic stress related to profession [ProQoL-Secondary Traumatic Stress; *t*(34) = 3.11, *p* = 0.01, *d* = 0.53], perceived stress [PSS; *t*(33) = 2.19, *p* = 0.04, *d* = 0.38], and self-compassion [SCS-SF; *t*(34) = −4.87, *p* < 0.001, *d* = −0.82; Figures 3–5]. Participants in the No Training group reported an increase in anxiety symptoms [GAD-7; *t*(12) = −2.50, *p* = 0.03] from pre-intervention to post-intervention, although the change was not significant after adjusting for multiple comparisons.

### Group differences in secondary outcomes

**FIGURE 6:**
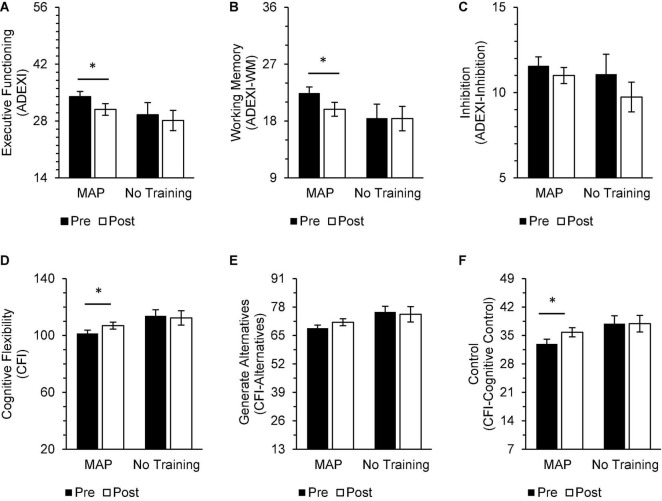
In the MAP group, post-intervention scores on measures of executive functioning [**(A)** ADEXI] and working memory [**(B)** ADEXI-WM], cognitive flexibility [**(D)** CFI], and cognitive control [**(F)** CFI-Control] were significantly improved from baseline. Participants in the MAP group did not report improvements in inhibition [**(C)** ADEXI-Inhibition] or ability to generative alternatives [**(E)** CFI-Alternatives]. There were no significant changes in self-reported cognitive functioning reported by participants in the No Training group. There were also no significant between-group differences in these domains at either timepoint. Asterisks indicate significant adjusted *p*-values. Total scores on the ADEXI range from 14 to 70, ADEXI-WM subscores range from 9 to 45, and ADEXI-Inhibition subscores range from 5 to 20.

Multivariate analyses of variance was applied to identify between-group differences in pre-intervention and post-intervention scores on cognitive measures, with no multivariate interaction (group × timepoint; *p* > 0.05). A series of independent samples *t*-test with FDR correction examined between group differences on cognitive measures at pre-intervention and post-intervention timepoints, consistent with the study’s *a priori* hypotheses. Pre-intervention scores on subjective estimates of cognitive flexibility [CFI; *t*(46) = −2.73, *p* = 0.01, *d* = −0.89], the ability to generate alternatives [CFI-Alternatives; *t*(46) = −2.69, *p* = 0.01, *d* = −0.87], and cognitive control [CFI-Control; *t*(46) = −2.30, *p* = 0.03, *d* = −0.75] were higher in the No Training group relative to the MAP Training group, but the difference was not significant after applying the FDR correction. Additionally, there were no significant differences between groups on these measures after the intervention (*p*s > 0.05). However, within the MAP group, post-intervention ratings on subjective measures of executive functioning [ADEXI; *t*(34) = 3.35, *p* = 0.01, *d* = 0.57], working memory [ADEXI-WM; *t*(34) = 4.37, *p* < 0.001, *d* = 0.74], cognitive flexibility [CFI; *t*(33) = −2.39, *p* = 0.02, *d* = −0.41] and cognitive control [CFI-Control; *t*(33) = −2.86, *p* = 0.01, *d* = −0.49] were significantly improved from baseline (Figure 6). There were no significant changes across time for teachers assigned to the No Training group.

**FIGURE 7:**
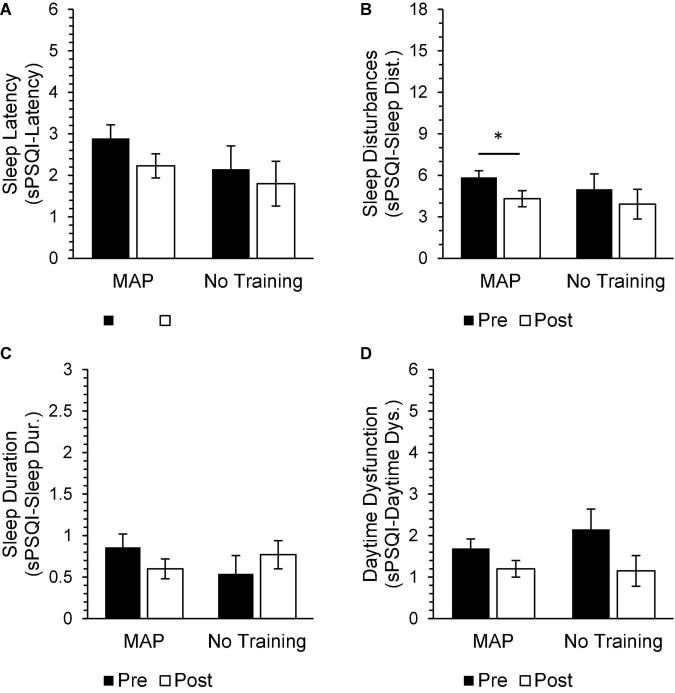
MAP participants reported a significant reduction in sleep disturbances post-intervention [**(B)** sPSQI-Sleep Disturbances]. MAP participants did not report any significant changes in other aspects of sleep quality, including sleep latency [**(A)** sPSQI-Latency], sleep duration [**(C)** sPSQI-Sleep Duration], and daytime dysfunction [**(D)** sPSQI-Daytime Dysfunction]. There were no significant differences between pre-intervention and post-intervention scores on health measures in the No Training group. There were also no significant differences between groups on these measures at either timepoint. Asterisks indicate significant adjusted *p*-values.

In general, subjective assessments of physical health did not change as a result of the intervention, although participants in the MAP Training group reported significantly fewer sleep disturbances [sPSQI-Sleep Disturbances; *t*(34) = 3.36, *p* = 0.01, *d* = 0.57] at intervention end (Figure 7).

## Discussion

MAP stands for “mental and physical” and MAP Training combines mental training with meditation and physical training with aerobic exercise to improve mental and physical health (Shors et al., 2014; Shors, 2021). The program has demonstrated efficacy in a number of studies with distressed populations, including men and women diagnosed with major depressive disorder (Alderman et al., 2016; Shors et al., 2017), young adult women who have experienced sexual trauma (Shors et al., 2018), mothers who were homeless (Shors et al., 2014), medical school students (Lavadera et al., 2020), and women living with HIV (Millon et al., 2022). The purpose of this pilot study was to examine the effects of an online version of MAP Training on teacher stress and related mental health outcomes experienced during the height of the COVID-19 pandemic. The findings suggest that MAP Training was beneficial, as delivered during the first summer of the pandemic, when most teachers were out of the classroom for the school year but were preparing to either go back into the classroom in the fall semester or teach virtually. Teachers who participated in the MAP Training program reported sizeable improvements (i.e., *d*s = 0.4–0.8) in mood (i.e., depressive symptoms), along with less anxiety, fewer ruminative thoughts, less perceived stress, and more self-compassion by intervention end. In addition, those who participated reported less work-related (secondary) traumatic stress when compared to those who did not participate. These positive results stand in contrast to those reported by teachers who did not participate and were instead assigned to a waitlist, some of whom reported increases in stress-related symptoms over the same time period.

Importantly, positive outcomes were observed despite the relatively brief duration of each session (1 h) and the course of the intervention (i.e., a maximum of two sessions per week over 6 weeks). Moreover, most participants did not attend all the sessions, averaging about four out of six live sessions and half of the recorded ones. The “effective” level of training is not inconsistent with previous studies. For example, women with a history of sexual trauma, reported fewer trauma-related thoughts and ruminations after two sessions per week over 6 weeks (Shors et al., 2018). In another study, women living with HIV reported similar outcomes after only one in-person session a week for 6 weeks (Millon et al., 2022). However, these finding do stand in contrast to those following many exercise-related interventions, which often depend on multiple sessions per week to produce sizeable improvements in mental health outcomes (Stathopoulou et al., 2006; Asmundson et al., 2013; Stanton and Reaburn, 2014). As a result, we do not claim that the positive outcomes reported here arise from the exercise component alone, but rather in response to the combination of meditation and aerobic exercise, especially when each activity is conducted one after another closely in time. Indeed, the combination of these two activities was reportedly more effective than either component alone in reducing trauma-related thoughts and ruminations, while at the same time improving self-worth (Shors et al., 2018).

In general, the present results suggest that the combination of FA meditation training and aerobic exercise may prevent or at least mitigate some of the mental health symptoms that arose during the height of the COVID-19 pandemic. Reported levels of anxiety tended to increase among teachers who did not participate in MAP Training over the 6 weeks. However, these changes were not significant after applying statistical correction, perhaps due in part to a less than desirable sample size in the control (No Training) group. However, the purpose of this study was to test whether an online intervention would support teacher mental health and well-being during the height of the pandemic. As a result, we could not continue to enroll participants beyond the summer months, as the impact of COVID-19 was evolving (due to vaccines, distance learning, etc.). Therefore, a large proportion of subjects were randomized to the MAP Training group.

### Rumination in teachers during the COVID-19 pandemic

Ruminations tend to be negative, about the past, and infused with some degree of blame or regret. After training, the K-12 teachers reported fewer of these thoughts, including brooding and depressive subtypes. There were no changes in reflective rumination, which is about the past and tends to be less negative. There are numerous theories and still some controversy surrounding rumination subtypes, but in general, depressive rumination is most tightly linked to changes in mood while brooding rumination appears to predict later depression (Treynor et al., 2003; Burwell and Shirk, 2007). In this study, intervention participants also reported fewer symptoms of depression. Thus, the effects of MAP Training on more detrimental aspects of rumination (i.e., brooding and depressive rumination) may be in part responsible for alleviating some of the depression reported by the teachers during the pandemic. It is also conceivable that this type of intervention, when practiced routinely, may help prevent some increases in depression that can arise during stressful life events.

In addition to depression, rumination is linked to other aspects of mental health and wellness, including anxiety, trauma-related cognitions, and even how someone interprets changes in their body. In a recent factor analytic study, ruminations accounted for much of the variance (>96%) in mental health outcomes acquired through many of the same self-report measures used here (Millon and Shors, 2021). Of course, there are similarities amongst the questions in these surveys and these similarities may account in part for the relationships. But nonetheless, these analyses have led us to suggest that rumination may serve as a proxy for overall mental health. All that being said, we do not know the neural or psychological mechanisms through which rumination may affect other health outcomes. A recent meta-analysis did identify neural networks that were especially engaged in people who are inclined to ruminate whereas other networks, especially those in the temporal cortex were less engaged (Zhou et al., 2020). Perhaps these later networks are becoming more engaged because of training. In theory, this would be consistent with the increase in executive function and cognitive flexibility reported here by those who engaged in MAP Training. Functional imaging studies are underway to test this hypothesis.

### Perceived vs. traumatic stress and distress

Stress has wide-spread effects on mental and physical health, many of which arise not only from exposure to the stressful life event itself, but also from one’s subjective appraisal of the experience (Lazarus and Folkman, 1984). Our results indicate that MAP Training lessened teachers’ perceived stress. This finding is especially meaningful given that teaching is recognized as a high-stress profession (Shernoff et al., 2011; Richards, 2012; Gallup, 2014; McCarthy, 2019), with job-stress increasing for many teachers after the onset of the COVID-19 pandemic. In addition to the direct experience of stress, teachers, like other helping professions, are susceptible to secondary traumatic stress (Hydon et al., 2015; Molnar et al., 2017). Secondary traumatic stress differs from vicarious trauma in that it involves emotional and behavioral reactions to secondary exposure to trauma as opposed to changes in cognitive schemas and beliefs (Jenkins and Baird, 2002). In this study, MAP Training led to a significant reduction in secondary traumatic stress. Therefore, MAP Training may lessen the risk and thus help prevent secondary traumatic stress and associated distress among teachers who may have been exposed to student trauma during the COVID-19 pandemic (i.e., Collin-Vézina et al., 2020; Kovler et al., 2021).

Despite reported improvements in mood, anxiety, and indicators of stress, we did not observe changes in teachers’ ratings of distress tolerance in this study. Interestingly, intolerance of uncertainty, a subcomponent of distress tolerance, has been identified as a unique predictor of future perceived stress (Bardeen et al., 2016). Thus, distress tolerance may have been somewhat impervious to the effects of the intervention given the general state of uncertainty and anxiety during the initial months of the COVID-19 pandemic (Twenge and Joiner, 2020). It is also important to note that distress tolerance is commonly conceptualized as a relatively stable, trait-like marker of psychopathology symptoms (Leyro et al., 2010). Moreover, some data suggest that standalone mindfulness interventions may lead to changes in behavioral distress (e.g., task persistence) but not perceived distress tolerance (e.g., Carpenter et al., 2019). Taken together, these data suggest that MAP Training may be effective in improving one’s current experience of distress as opposed to their perceived ability to relate to symptoms (i.e., distress tolerance).

### Subjective estimates of cognition and physical health during the COVID-19 pandemic

Virtual delivery of MAP Training during the pandemic also had a positive impact on teachers’ subjective ratings of cognitive functioning. Treatment adherent participants reported significant improvements in working memory and executive functioning of medium effect size after 6 weeks of MAP Training. Additionally, participants who adhered to the program reported significant improvements in cognitive flexibility and control following training, whereas no such change was observed among waitlist control participants. The impact of MAP Training on self-reported cognition in this study is consistent with previous research on the cognitive effects of mindfulness meditation interventions (Chiesa et al., 2011) as well as considerable data demonstrating a positive link between exercise and brain function (i.e., Ludyga et al., 2020). And as noted, MAP Training has been associated with an increase in amplitude of the early components of the evoked response during the Flanker task, which engages neural processes related to executive function and cognitive control (Alderman et al., 2016).

Aerobic exercise alone has numerous effects on brain function, including increases in vascular growth, neurogenesis in the hippocampus, as well as the release of growth factors such as BDNF (i.e., van Praag et al., 1999; Piepmeier and Etnier, 2015; Phillips, 2017; Vivar and van Praag, 2017; Liu and Nusslock, 2018). Meditation has also been linked with increases in hippocampal volume among those who practice meditation regularly (Luders et al., 2013) and reductions in hippocampal atrophy in those with mild cognitive impairment (Wells et al., 2013), possibly through effects of synaptogenesis, angiogenesis and neurogenesis. In fact, MAP Training was developed for humans based on preclinical studies suggesting that mental training with effortful learning procedures increases neurogenesis in the adult hippocampus (Gould et al., 1999; Leuner et al., 2004; Curlik and Shors, 2011; Shors, 2021). Others have reported that a combination of spontaneous learning during environmental enrichment when preceded by aerobic exercise is especially neurogenic (Fabel et al., 2009). Therefore, the combination of mental (i.e., FA meditation) and physical (i.e., aerobic exercise) training coupled with enhanced mood may produce cognitive change in humans through mechanisms of hippocampal plasticity.

The combination of mental and physical training had a positive impact on teachers’ physical wellbeing, specifically on the quality of sleep. Participants reported fewer sleep disturbances including fewer nighttime awakenings, breathing difficulties and nightmares relative to their reports before training. There were no such changes in the waitlist control condition. Sleep quality is an important predictor of subjective well-being (Reid et al., 2006; Peach et al., 2016; Wickham et al., 2020) and demonstrates a bidirectional relationship with depression and anxiety symptoms (Alvaro et al., 2013). Given the increased prevalence of poor sleep quality during the COVID-19 pandemic (i.e., Gupta et al., 2020; Pinto et al., 2020; Hyun et al., 2021), interventions that improve sleep, even indirectly, are especially needed.

MAP Training does not necessarily impact mental health through direct changes in cardiovascular activity. For example, we observed no change in heart-rate variability and related measures of sympathetic nervous system activity after women with HIV completed 6 weeks of training, despite robust decreases in ruminative and trauma-related thoughts (Millon et al., 2022). However, training did increase the volume of oxygen consumed in women with physical complaints such as addiction and malnutrition (Shors et al., 2014). In contrast, the current participants were relatively young high-functioning adults. It is likely that individual differences in physical health prior to training are important (i.e., the potential range for change) as well as the length of the intervention, which is relatively short per session (1 h) and over its course (6 weeks).

### Limitations and considerations

There are several limitations to this research. First, our results were limited by a less than desirable sample size and suboptimal adherence, with most participants completing about four out of six of the live sessions, and half of the weekly recorded sessions. High rates of attrition are commonly observed in studies of virtual and web-based interventions (Eysenbach, 2005; Melville et al., 2010). In this study, dropout rates may have been exacerbated by an overall increase in anxiety, depression, and stress in the general population during the COVID-19 pandemic (e.g., Xiong et al., 2020), and especially among teachers (e.g., Aperribai et al., 2020; Anderson et al., 2021; Ozamiz-Etxebarria et al., 2021). Indeed, data from reviews indicate that the COVID-19 pandemic has resulted in substantial declines in participant enrollment in clinical trials and research studies (Sathian et al., 2020). Additionally, we began recruitment during the beginning of summer when most teachers were out or soon to be out of the classroom. And thus, burnout and workplace fatigue may have lessened their willingness to participate (Pressley, 2021). Because the conditions of the pandemic were constantly changing, we could not continue to recruit once they had returned in the fall to the classroom. Yet, despite these restrictions, we observed significant and positive effects of MAP Training on subjective estimates of mental health and well-being.

The MAP Training intervention was delivered virtually to accommodate stay-at-home orders and remote working conditions during the COVID-19 pandemic. Importantly, the teachers reported a high level of engagement and their average heart rate during the physical training was 130 beats per minute, suggesting that they were exercising at an intensity consistent with aerobic exercise. Nevertheless, there were certain limitations to the virtual format. For example, the participants’ cameras were disabled during the live sessions to protect their privacy. (And several participants stated beforehand that they did not wish to turn on the camera). Therefore, it is not known whether participants were fully adhering to the MAP Training intervention. Additionally, variability in internet and technology literacy and quality may have interfered with their engagement. These potential barriers have been documented in prior studies of virtual interventions (e.g., Borghouts et al., 2021) and have yet to be adequately addressed.

Finally, we recruited teachers within the local tri-state area because of similarities in the impact of COVID-19 in this region. At the time of the study, COVID-19 cases were among the highest in the country and residents were facing statewide travel restrictions, mask mandates, limits on social gatherings, and supply shortages. Thus, we cannot be certain that our findings would generalize to other populations of teachers within or outside of the United States.

### Implications and future directions

Overall, findings from this pilot study suggest that 6 weeks of virtual MAP Training can lead to positive changes in select measures of mental health, especially those related to mood, negative thinking, and overall well-being. Even prior to the COVID-19 pandemic teachers were affected by high levels of occupational stress (Gallup, 2014), which has been associated with poor mental health outcomes, including depression and anxiety (Besse et al., 2015; Jones-Rincon and Howard, 2019). Moreover, workplace burnout has been closely linked with depression (Ahola et al., 2014), with some studies suggesting significant overlap between the two constructs (Schonfeld and Bianchi, 2016). Therefore, interventions that target psychological distress, such as MAP Training, may help to reduce burnout and subsequent rates of attrition among teachers, even under normal working conditions. The mental and physical health benefits of meditation and aerobic exercise have been demonstrated independently in a variety of populations (e.g., Warburton, 2006; Gillison et al., 2009; Chiesa et al., 2011; Keng et al., 2011; Kvam et al., 2016; Stubbs et al., 2017; Creswell et al., 2019), including teachers (e.g., Crain et al., 2017; Klingbeil and Renshaw, 2018; Zarate et al., 2019; Abós et al., 2021). The present results highlight the potential benefit of combining these activities together in one intervention to alleviate stress and promote well-being in teachers. They further suggest that the virtual delivery of interventions such as MAP Training are effective in improving mental health and mitigating the impact of stressful life events, such as occurred during the coronavirus pandemic.

## Data availability statement

The raw data supporting the conclusions of this article will be made available by the authors, without undue reservation.

## Ethics statement

The studies involving human participants were reviewed and approved by Rutgers University Institutional Review Board. The patients/participants provided their written informed consent to participate in this study.

## Author contributions

DD and TS implemented the research. DD performed the analyses. All authors devised the project, contributed to interpretation of results and writing of the manuscript, and approved the final version.
